# Mini-Perc for Renal Stones—A Single Center Experience and Literature Review

**DOI:** 10.3390/diagnostics13061083

**Published:** 2023-03-13

**Authors:** Victor-Mihail Cauni, Mihai Dragutescu, Bogdan Mihai, Gabriel-Petre Gorecki, Liana Ples, Romina-Marina Sima, Cristian Persu

**Affiliations:** 1 Department of Urology, Colentina Clinical Hospital, 020125 Bucharest, Romania; 2 Department of Anesthesia and Intensive Care, CF2 Clinical Hospital, 011464 Bucharest, Romania; 3 Faculty of Medicine, Titu Maiorescu University, 031593 Bucharest, Romania; 4 Department of Obstetrics and Gynecology, “Carol Davila” University of Medicine and Pharmacy, 050474 Bucharest, Romania; 5 “Bucur” Maternity, Saint John Hospital, 012361 Bucharest, Romania; 6 Department of Urology, “Carol Davila” University of Medicine and Pharmacy, 020021 Bucharest, Romania

**Keywords:** mini-perc, kidney lithiasis, minimally invasive nephrolithotomy

## Abstract

Aim: The aim of this study was to analyze the outcomes of miniaturized nephrolithotomy (mini-perc) in the management of renal stones with a diameter smaller than 20 mm. Materials and Methods: We retrospectively reviewed the records of 102 patients who underwent mini-perc between March 2015 and March 2020 in our department. The primary objective was the stone-free rate, but we also analyzed the retreatment rate, complications, hospital stay, operative time and reduction in hemoglobin level. All these patients had this technique as their first-line treatment, in a prone position, using a 16 Fr sheath size. Data were compared to a series of patients from the literature, treated with conventional PCNL. Results: The patients had calculus limited to either a single calyx or just extending to the renal pelvis, and stone size was less than 20 mm in its maximal dimension. The intrarenal stone location was in the upper calyx in 7 cases, middle calyx in 20 cases and lower calyx in 46 cases, and there were 29 patients with renal pelvis stone. The male to female ratio was 1.5:1, and the median age was 48.4 years. The average stone size was 17.4 mm in diameter (ranging between 9 and 20 mm) and all cases underwent Ho-YaG laser lithotripsy, ballistic energy and combined ultrasonic and ballistic lithotripsy. At the end of the procedure, an antegrade double J stent was placed under fluoroscopy for a maximum of 2 weeks in 42 cases, while 9 cases needed a nephrostomy tube 12–14 F. A total of 51 cases were totally tubeless. Our median operative time was 61 min (ranging from 35 to 75 min). The median hospitalization stay was 3.8 days. The stone free rate was 90.1% after one procedure, only nine (8.8%) cases needed a “second look” flexible ureteroscopy, and the final stone-free rate was 98% (absence of detectable calculi on ultrasound, KUB or non-contrast CT scan). The overall complication rate was 6.86% (Clavien classification I—57.14%; II—28.5%; III—14.2%), while no Clavien IV or V complications were reported. No patient required a blood transfusion, and mean hemoglobin loss was 0.81 mg/dL. Overall, our results are better than similar data for conventional PCNL in the literature. Conclusions: The “mini-perc” technique is an effective procedure for the treatment of the renal lithiasis that is less or equal to 2 cm. The results demonstrated that this minimally invasive technique is associated with a higher stone-free rate and minimal complications.

## 1. Introduction

Kidney stones, an entity known to humanity since antiquity, challenged the imagination of both doctors and general population in the quest for finding better diagnostic methods and better treatments. By better, we generally mean efficacy, but complications and time spent during surgery and overall time spent in the hospital remain important. Given that kidney stones affect roughly 1 in 8 men and 1 in 16 women during their lifetimes, the topic becomes very relevant in terms of the number of patients. Current urolithiasis guidelines recommend PCNL as the first-line treatment in cases with kidney stones larger than 20 mm in the diameter. For the size interval between 10 and 20 mm, more options are available: ESWL, PCNL and RIRS [1,2], and this relative overlapping leaves place for debate while the final choice remains with the surgeon, based on his experience and the technical possibilities of the hospital.

Percutaneous nephrolithotomy was first described as a technique in 1976 by Ferstron and Johannson [3]. After almost two decades, Jackman and Helal proposed a miniaturized access sheath (13 F) for a percutaneous approach in pediatric patients. It was only a small step to introduce smaller size instruments to the adult population as well. Since then, technical progress nowadays permits a better image, a greater variety of lithotripsy sources, better irrigation systems and more reliable instruments. Despite some initial skepticism [4], in the end, the mini-perc technique imposed itself as the standard of care in the treatment of urolithiasis [5] in many cases and an excellent alternative in many others.

By utilizing miniaturized instruments, the industry created the prospective for lower complication rates in comparison with classic PCNL, particularly by diminishing parenchymal injury created by the access sheath. A standard size mini-perc access sheath varies between 14 and 20 F [6] or about 3 to 4 cm. The main limit and risk factor of percutaneous renal surgery is the blind access through the parenchyma, potentially leading to uncontrolled bleeding, lesions of the collecting system and permanent damage to the kidney. While mini-perc does not eliminate completely those risk factors, a lower diameter of the access tract is demonstrated to significantly reduce the incidence of post-operative complications while maintaining efficacy, particularly due to the improved energy sources used for lithotripsy and improved imaging during the intervention. While monitoring data from the international literature, we observed two main lines of evolution: for larger stones, standard PCNL tends to be the most used approach, while for smaller stones, retrograde flexible ureteroscopy gained a lot of trust from the urological community. There are obvious advantages of flexible ureteroscopy, while the overall operating time remains the main drawback of this procedure. Based on the significant experience we acquired with standard PCNL and the ease of shifting to mini-perc in our daily practice, we consider that this technique deserves more attention.

The aim of this study was to analyze the outcomes of miniaturized nephrolithotomy (mini-perc) in the management of renal stones with a diameter below 20 mm in patients from our urology department. The data we gathered were compared to similar reports from the literature. We also propose a brief literature review aiming to describe the current state of the art in the field of minimally invasive kidney stone surgery.

## 2. Materials and Methods

We designed a study which retrospectively reviewed the records of the patients who underwent mini-perc nephrolithotomy between March 2015 and March 2020 in our urology department. Our center has significant experience in endourology, being an early adopter for many new techniques. At the same time, we have more than 20 years of experience with PCNL and different types of energy sources for stone fragmentation.

The primary outcome objective of our work was the stone-free rate. At the same time, we analyzed the retreatment rate, surgical complications, total hospital stay, total operative time and reduction of hemoglobin level, all defined as complications of the procedure.

All our patients signed a standardized informed consent form prior to their surgery and the current standard-of-care diagnostic protocol and treatment were used. Before the study, Ethics Committee approval from our hospital was requested and obtained to review and analyze the data.

The evaluation protocol included a detailed medical history and physical examination of the patient. Laboratory tests including urinalysis, urine culture, complete blood count, serum biochemistry and coagulation test. Imaging of the urinary tract started with routine ultrasonographic examination, then a kidney, ureter and bladder X-ray (KUB) was performed. Functional evaluation consisted of intravenous urography (IVU) or a CT scan with a contrast medium, performed in all patients. While we prefer the CT scan over the IVU, in some cases it could not be conducted, for non-medical reasons, such as insurance aspects or technical difficulties which made the CT scanner unavailable for a longer period of time.

In the case of positive urine cultures, our patients received the appropriate antibiotics based on the sensitivity test but this did not lead to cancelation of the intervention in any case, although surgery in some patients was delayed for a few days.

The data obtained were compared against a meta-analysis from the literature, evaluating patients treated with conventional PCNL with similar stone features. We looked at the stone-free rate, operative time, blood loss and Clavien–Dindo complications rate.

Clavien–Dindo classification aims to rank surgical complications in an objective and reproductible manner. For this purpose, there are five grades, with Grade V being the most serious. Grade I includes any deviation from the normal postoperative course, with no need for pharmacological, surgical or other type of treatment. Grade II requires medication or blood transfusion to treat the complications. Grade III means surgical intervention to correct the complication. As per our study, the second look intervention is not considered a complication but a normal evolution of the postoperative course. Grade IV includes any life-threatening complications requiring intensive care management. Grade V means the death of the patient.

The patients we included had this technique as their first-line treatment, in a prone position, using a 16 Fr sheath size, according to our standardized procedure described below. Patients with stones fulfilling the size criterion for our study but who had previous surgery for kidney stones were not included in this evaluation. This was the same for patients who were not treated because of anesthesiological concerns or based on their personal decision to refuse the percutaneous approach.

### Mini-Perc Technique

The procedure was conducted under spinal or general anesthesia. The patient was positioned in the lithotomy position, then a 5-Fr ureteral catheter was inserted through the ureter. The patient was then moved to a prone position and the calyceal system morphology was evaluated via fluoroscopy using a contrast medium. Access was gained to the desired calyx using an 18 G needle under fluoroscopy unit monitoring. As a rule of thumb, the approach of the kidney, dilation and sheath insertion are very similar to regular PCNL. Access to the collecting system was achieved through the lower posterior calyx; alternatively, the calyx with the largest stone burden or the calix promising access to most of the stones were targeted [7,8,9]. As an alternative technique, renal access was obtained under ultrasonographic guidance, still in a prone position, in some cases. This did not have any impact on the operative technique itself or the overall duration of the intervention.

**Figure 1:**
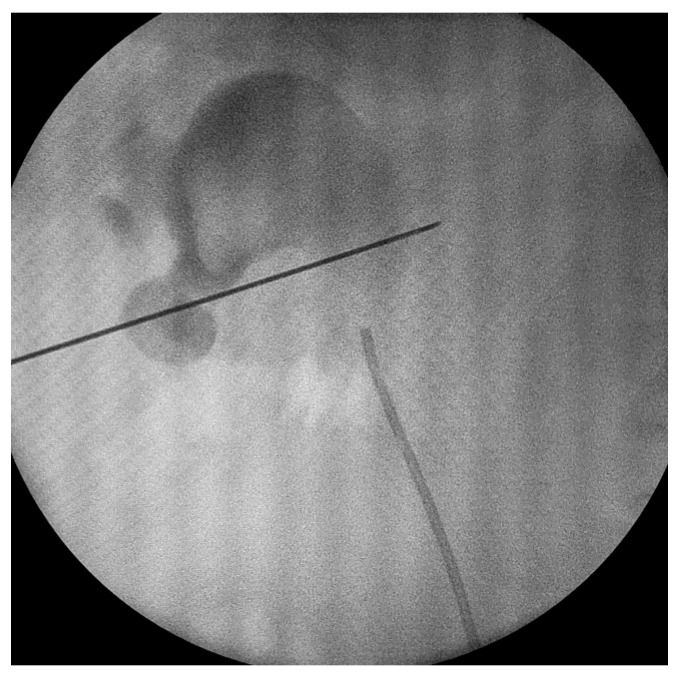
Targeting of the puncture site.

**Figure 2:**
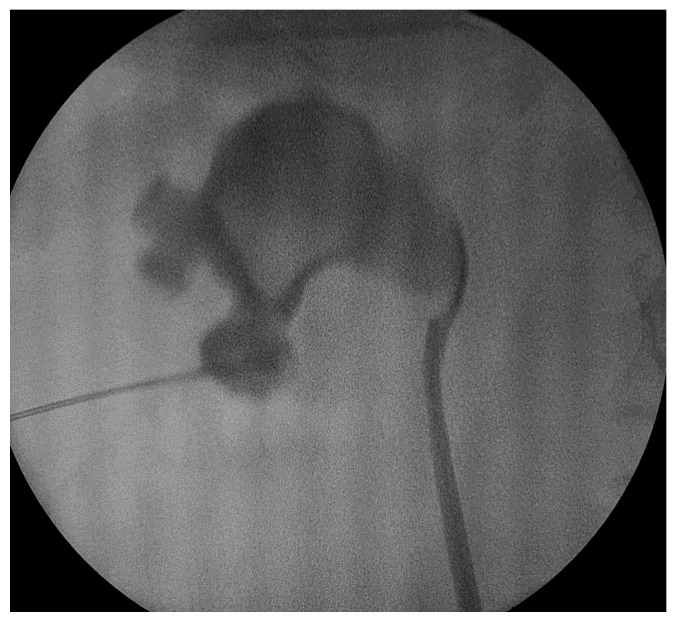
Percutaneous renal access.

After that, a 0.035-inch hydrophilic guide wire was inserted into the collecting system. Dilation was performed using Amplatz dilators, and a 16 Fr Amplatz sheath was inserted. We used a 15.9 F rigid nephroscope and stone fragmentation was performed using Ho-YaG laser lithotripsy (350 or 550 μm fiber), ballistic energy or combined ultrasonic and ballistic lithotripsy (Figure 1 and Figure 2). Stone fragments were removed in the next step using the so called “vacuum effect” or using grasping forceps. The “vacuum effect” is based on a hydro-dynamic phenomenon which takes place during low-pressure, continuous-flow PCNL. Stone fragments located in line with the irrigation channel of the nephroscope were washed by the flow of water [10,11]. Some fragments were not removed with this technique so they needed extraction using a 1.9 F stone basket or tri-radiate graspers. After all fragments were removed, the collecting system was inspected visually and then evaluated with a contrast medium, under fluoroscopy, in order to identify residual stones and fragments or perforations.

When stone processing was over, in the case of pelvicalyceal perforation, if residual fragments were present, or according to the operator’s choice, an antegrade double J stent was placed under fluoroscopy. Alternatively, in some cases, a nephrostomy tube was left in place. Nephrostomy tubes were removed after 48 h if no second-look surgery was required, and double J stents were removed via the retrograde approach, usually after about two weeks.

As a general impression, based on our significant experience with standard PCNL, the adoption of mini-perc came easily and with no major barriers, so we consider that this step will be the same for other centers exploring the possibility of introducing this less invasive technique.

## 3. Results

The study included 102 patients treated with mini-perc during the evaluated period in our department. The demographic characteristics of the patients are presented in Table 1.

**Figure 3:**
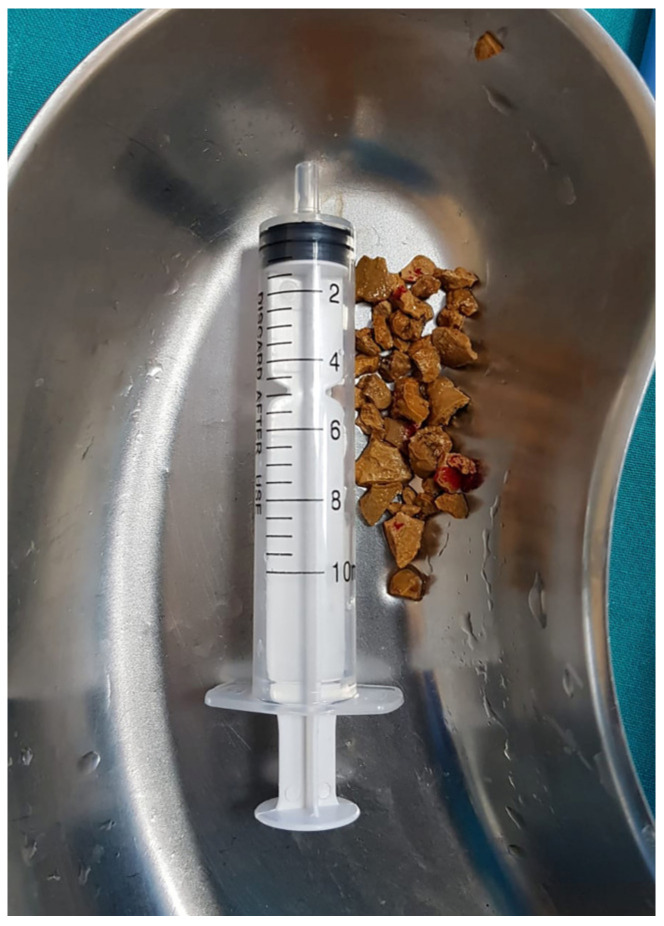
Stone fragments after mini-perc technique.

The stone was located in either one single calyx or just extending to the renal pelvis and the stone size was less than 20 mm in its maximal diameter. The intrarenal stone location was as follows: upper calyx in 7 cases, middle calyx in 20 cases and lower calyx in 46 cases, while 29 patients had a renal pelvis stone. The male to female ratio was 1.5:1, and the mean age was 48.4 years old. The stone size was measured on the longest axis on preoperative imaging. The mean stone size was 17.4 mm in diameter (ranging between 9 and 20 mm) (Figure 3). In cases where a CT scan was available, we measured a median value of about 900 HU, ranging from 740 to 1290 HU.

The median operative time was 61 min (range 35–75 min). The median hospitalization stay was 3.8 days. This is in part due to a practical limitation on our side, because patients need to be admitted to the ward before blood and urine can be sampled. We speculate that should this not be necessary, and the surgery could be completed on the day of admission, the mean hospital stay would most likely decrease to 2.8 days. The stone-free rate was 90.1% after one procedure, with only nine (8.8%) cases needing a “second look” flexible ureteroscopy, and the final stone-free rate was 98%, defined as the absence of detectable calculi on ultrasound, KUB or contrast or non-contrast CT scan.

The overall complication rate was 6.86% (Clavien classification I—57.14%; II—28.5%; III—14.2%), with no Clavien IV or V complication. No patient required a blood transfusion, while the median hemoglobin loss was 0.81 mg/dL.

The operative time was measured from anesthesia until the placement of the double J stent or nephrostomy tube, when required, and the median operative time was 61 min (range 35–75 min).

In 42 cases, a double J stent was inserted at the end of the procedure, while 9 cases were offered a nephrostomy tube 12–14 F. The other 51 cases were totally tubeless, and the figures seem to be lower than similar cases where standard PCNL is performed. Since this was not an objective of our study, we did not evaluate this particular aspect in detail. An overview of the main surgical parameters is summarized in Table 2.

The stone composition evaluated on post-surgical spectroscopy analysis included 49 monohydrate calcium oxalate, 38 dihydrate calcium oxalate, 13 calcium phosphate and 3 uric acid calculi.

In our study, the mean hospital stay was relatively short, given that the main predictive factor for reducing hospital stay is nephrostomy tube removal. The stone-free rate was evaluated after Somani et al. classification, meaning the absence of residual fragments visible on ultrasound, KUB or CT. Accordingly, stone-free rate in our group was in line or better when compared to similar studies from the international literature.

The overall complication rate in our study was generally lower or comparable with similar studies from experienced centers or doctors. We speculate that this aspect correlates more with the general experience of the center, given that even for standard PCNL, our complication rate is by no means higher than what is reported in other large-scale studies, while remaining lower than what is reported in studies from smaller, non academic centers.

Our data are in line with the latest results from the international literature. Complication rates from our study have many explanations. We believe that diligent case selection based on stone size, location, etc., has a major impact on the outcomes of the procedure. In seems obvious that a center where all the methods are available will use PCNL or mini-perc where it is really the best option.

In our opinion, the approach type is the most important aspect in percutaneous interventions. In this way, mini-invasive nephrolithotomy offers safety and comfort by choosing single-stage dilators for which fewer technique steps are needed, in comparison with classic PCNL where Amplatz serial dilators are usually used. We consider that multiple benefits can arise from this approach: image quality, multiple lithotripsy methods, lower renal parenchymal damage, etc.

Because the learning curve is short for urologists with previous experience with percutaneous procedures and there are a lot of advantages for this technique, we believe that surgeons performing classic PCNL and retrograde and antegrade ureteroscopy should consider minimally invasive nephrolithotomy for selected patients, if the relevant instruments are available or can be acquired.

## 4. Discussions

An analysis of the up-to-date urological literature shows that “stone-free” is a variable notion. No less than seven different definitions being identified after 249 studies, the most frequent one being “total stone absence”. In addition, only 68% studies mention this aspect, meaning that a third of them do not define the term “stone-free” [12], though they probably mean the same thing.

Moreover, imaging methods utilized for assessing this status are different: the most frequent one is KUB (28%), followed by the combination of KUB and renal ultrasonography (22%). Data from the literature show that the stone-free rate on post-operative imaging is generally lower than the rate based on intraoperative evaluation at the end of the procedure, and this should be considered as some fragments might be “hiding” during surgery [11]. This has an impact on the post-surgical discussion with the patient, when a reserved attitude might be recommended, since it is possible that further evaluation will diagnose residual fragments which were not identified at the end of the procedure. We recommend that a stone-free status should be communicated to the patient only after post-operative imaging and should not be based only on intra-operative findings.

The most frequent therapeutic approaches were PCNL (47%), ESWL (33%) and ureteroscopy (13%). Thereby, most authors have reached the conclusion that a better standardization of stone-free rates is needed in the literature [13] and we support that, aiming for a better comparison between different series of patients from different centers. Alternatively, when comparing different studies, it is important to check the way each study defines the stone-free rate, otherwise the results might not be truly comparable [14].

Comparing our data with a meta-analysis from the international literature [15], we report a stone-free rate of 98% (after the second procedure, when needed), compared to 89.9% after standard PCNL in other studies. Average blood loss in our study was 0.81 mg/dL, significantly lower that the 1.2 mg/dL reported in the meta-analysis. Operative time in our series was 61 min, while for standard PCNL, it is reported at 79 min. The complications rate using the Clavien–Dindo scale was 6.86% in our study and 10.5% in the literature when evaluating conventional PCNL.

In a study conducted on 351 patients with urolithiasis treated with PCNL with a single-access tract, the success rate (defined as stone-free rate-SFR) was 76% and the main factors that influenced this result were the stone size, upper calix localization and prior ESWL interventions. The high SFR is attributed to a good case selection—calculi lower than 20 mm and an accessible location combined with a normal anatomy. As a matter of fact, similar results were achieved by Abdelhafez et al., (96.7%) for simple lithiasis [16], and a 94.3% stone-free rate by Resorlu B et al., on 106 patients [17] and many others mentioned below (Table 3).

A review of the recently published papers shows a low overall complication rate, even in groups of more than 1000 patients. This confirms the major benefits of minimally invasive surgery but should not preclude the development of even more advanced techniques, both in terms of efficacy and safety (Table 4).

In Abdelhavez and his colleague’s opinion, mini-PCNL proved more effective in the treatment of smaller stones, in general under 20 mm, rather than for stones larger than 20 mm (SFR 90.8% vs. 76.3%), while the operative time increases with size.

If mini-perc is compared to RIRS used for large renal calculi (20–30 mm), the studies prove that mini-PCNL can lead to a significantly higher SFR (96.6% vs. 71.4%) [21,22]. Similar outcomes of the two methods in terms of effectiveness is reported in the treatment of smaller stones [23,24]. We speculate that this is because of more powerful energy sources used in mini-perc and better view and movement options compared to RIRS, where the smaller size of the instruments and the limited mobility required by the endoscopic approach compromise, in part, the efficacy of the technique.

The main advantage of mini-perc over RIRS is more obvious for the approach of large impacted proximal stones (≥15 mm), with SFR rates of 93.3% for percutaneous vs. 41.4% for RIRS [25]. The authors attribute this to the same aspects we mention above.

In the urolithiasis treatment guidelines, the treatment recommendation for kidney stones substantially depends on stone size and location. However, many authors stated that the total number of stones negatively affected all procedures’ outcomes, including SWL, flexible URS and PCNL. Ackermann et al. found that the stone number was better correlated with the procedure success than the stone burden [26].

During intraoperative stone fragmentation or SWL, multiple small stones can easily migrate and escape from laser or shock waves. Kanao et al. emphasized that focusing on one large stone was easier than targeting multiple small stones with the same stone burden [27]. This probably has to do with the complexity of the internal kidney architecture and the limits of percutaneous access.

Age is considered a factor that influences hospital stay and complication rate rather than the overall success rates, as described in a recent study in which authors demonstrate that older age is associated with longer hospitalization, mainly because more complications occur. Nevertheless, the overall stone-free rate is similar across all age groups [28].

The standard definition of complications was used, as any adverse event occurring within 30 days, and quantified by the modified Clavien–Dindo classification system. A large scale study from the United States describes the characteristics of sepsis and acute pyelonephritis associated with upper urinary tract stones, setting a baseline for better managing these patients in the future [29].

Not long ago, there was a general belief that a small diameter of the channel can increase intrarenal hydrostatic pressure for a better visualization and thus a greater complication rate, especially for sepsis, but Hu. et al., showed that in the case of mini-invasive nephrolithotomy (14, 16 and 18 F), intrarenal pressure should not exceed 30 mm Hg, the threshold from which extravasation is expected [30].

A recent study by Li et al. [17] describes the combination of the percutaneous approach with retrograde ureteroscopy for complex renal stones. Since this technique is not explicitly mentioned by the current guidelines, the authors did a thorough review of the literature and meta-analysis, being able to demonstrate the superiority of this combined approach in terms of stone-free rate, operative time, complications and the number of access tracts, an important limit of percutaneous surgery. The authors conclude that for complex stones, the combined access is superior to PCNL alone [31].

**Table 4:** Complication rate.

Reference	Total, *N*	Complication Rate, %
Li et al. [17]	4760	0.86 (major)
Hu et al. [30]	1368	20
Gu et al. [31]	611	6
Xu et al. [32]	1200	7.6
Cirillo et al. [33]	209	7
Long et al. [18]	163	0 (major)
Del Giudice et al. [29]	13,984	19/26
Zeng et al. [20]	8868	16.3

Another recent study looked at the potential factors which might contribute to the suboptimal results of PCNL. The authors demonstrate that staghorn calculi and stones in multiple locations are the main risk factors for failing to achieve stone-free status while hydronephrosis increases the risk of complications and longer hospital stays, mainly because of the need for internal or external drainage of the collecting system after the intervention. Large stone volume and staghorn calculi significantly increased the operative time. Based on their statistics, the authors proposed algorithms for making informed decisions and setting realist expectations for both doctors and their patients [32].

Modern times bring along the popularization of innovative, complex technology, making it possible for patients to benefit from their smartphones to gather information from reliable sources on symptoms, prophilaxy and treatment options, and, at the same time, to calculate risks associated with their condition [33]. Artificial intelligence models will definitely change the future of urinary stone treatment, potentially reducing the need for surgical treatments.

The limits of our study include the retrospective analysis of data, a relatively small sample size and the lack of an active comparator. We mitigate this considering that a prospective, randomized study would be much harder to accept by the patients and ethics committee while an active comparator for this type of surgery does not exist. Another limit of our work is the different energy sources we used for lithotripsy; however, by using the best available method in each case, we think that there is no bias here. The procedures were performed by three urologists, and that might be seen as a limit; however, we consider that this way we truly report the experience of our center rather than a personal experience with a technique. We consider that the retrospective nature of our work eliminates the bias of potentially thinking ahead, and during surgery, thinking about the results we would like to obtain, and see this as a plus. Our center has a significant experience performing “classic” PCNL and we analyzed our results taking into account this experience.

## 5. Conclusions

The “mini-perc” is an effective procedure for the treatment of renal lithiasis with the largest diameter of up to 2 cm. Our results demonstrate that this minimally invasive technique is associated with a high stone-free rate and lower incidence of complications, while the learning curve is reasonably short for urologists with previous experience in endoscopic procedures. The review of the literature and our results also demonstrate that the general complication rate is not predictable by external factors other than the age of the patient.

## Figures and Tables

**Table 1:** Patients’ demographic and preoperative data.

Variable	Value
Number of patients	102 (62 males, 40 females)
Male-female ratio	1.5:1
Age (years)	Median 48.4 y.o.
Stone size, mm, median	17.4 (9–20)
Location, *n* (%)	
Upper calyx	7 (6.8%)
Middle calyx	20 (19.6%)
Lower calyx	46 (45%)
Renal pelvis	29 (28.4%)
Number of procedures	102

**Table 2:** Overview of surgical parameters in our series.

Surgical Parameter	Value
Stone-free rate	90.1% after one procedure 98% after second look (where needed)
Operative time	61 min (median)
Clavien–Dindo complications	6.86%
Blood loss	0.81 ng/mL (median)
Stenting of the collecting system	Double J stent—42 cases Nephrostomy—9 cases No stenting—51 cases

**Table 3:** Stone-free rate in mini-perc.

Authors	Year	*n* (Patients)	SFR% Init/Final
Nagele et al. [8]	2008	29	96.5/100
Li et al. [17]	2009	3610	89/91
Long et al. [18]	2013	163	95.7/-
Kirac et al. [19]	2013	37	91.9/97.2
Zeng at al. [20]	2013	12,482	78.6/94.8

## Data Availability

The data presented in this study are available on request from the corresponding author. The data are not publicly available due to internal regulations of the hospital.
